# Incentivizing appropriate malaria case management in the private sector: a study protocol for two linked cluster randomized controlled trials to evaluate provider- and client-focused interventions in western Kenya and Lagos, Nigeria

**DOI:** 10.1186/s13012-020-01077-w

**Published:** 2021-01-20

**Authors:** Aaron M. Woolsey, Ryan A. Simmons, Meley Woldeghebriel, Yunji Zhou, Oluwatosin Ogunsola, Sarah Laing, Tayo Olaleye, Joseph Kipkoech, Bomar Mendez Rojas, Indrani Saran, Mercy Odhiambo, Josephine Malinga, George Ambani, Emmah Kimachas, Chizoba Fashanu, Owens Wiwa, Diana Menya, Jeremiah Laktabai, Theodoor Visser, Elizabeth L. Turner, Wendy Prudhomme O’Meara

**Affiliations:** 1 Clinton Health Access Initiative, Boston, MA USA; 2 Duke Global Health Institute, Duke University, Durham, NC USA; 3 Department of Biostatistics and Bioinformatics, Duke University, Durham, NC USA; 4 Clinton Health Access Initiative, Lagos, Nigeria; 5 Academic Model Providing Access to Healthcare, Moi University, Eldoret, Kenya; 6 School of Social Work, Boston College, Boston, MA USA; 7 Clinton Health Access Initiative, Abuja, Nigeria; 8 College of Health Sciences, Moi University School of Public Health, Eldoret, Kenya; 9 College of Health Sciences, Moi University School of Medicine, Eldoret, Kenya; 10 Department of Medicine, Duke University, Durham, NC USA

**Keywords:** Malaria, Private sector, ACT, RDT, Subsidy, Subsidies

## Abstract

**Background:**

A large proportion of artemisinin-combination therapy (ACT) anti-malarial medicines is consumed by individuals that do not have malaria. The over-consumption of ACTs is largely driven by retail sales in high malaria-endemic countries to clients who have not received a confirmatory diagnosis. This study aims to target ACT sales to clients receiving a confirmatory diagnosis using malaria rapid diagnostic tests (mRDTs) at retail outlets in Kenya and Nigeria.

**Methods:**

This study comprises two linked four-arm 2 × 2 factorial cluster randomized controlled trials focused on malaria diagnostic testing and conditional ACT subsidies with the goal to evaluate provider-directed and client-directed interventions. The linked trials will be conducted at two contrasting study sites: a rural region around Webuye in western Kenya and the urban center of Lagos, Nigeria. Clusters are 41 and 48 participating retail outlets in Kenya and Nigeria, respectively. Clients seeking care at participating outlets across all arms will be given the option of paying for a mRDT—at a study-recommended price—to be conducted at the outlet. In the provider-directed intervention arm, the outlet owner receives a small monetary incentive to perform the mRDT. In the client-directed intervention arm, the client receives a free ACT if they purchase an mRDT and receive a positive test result. Finally, the fourth study arm combines both the provider- and client-directed interventions. The diagnosis and treatment choices made during each transaction will be captured using a mobile phone app. Study outcomes will be collected through exit interviews with clients, who sought care for febrile illness, at each of the enrolled retail outlets.

**Results:**

The primary outcome measure is the proportion of all ACTs that are sold to malaria test-positive clients in each study arm. For all secondary outcomes, we will evaluate the degree to which the interventions affect purchasing behavior among people seeking care for a febrile illness at the retail outlet.

**Conclusions:**

If our study demonstrates that malaria case management can be improved in the retail sector, it could reduce overconsumption of ACTs and enhance targeting of publicly funded treatment reimbursements, lowering the economic barrier to appropriate diagnosis and treatment for patients with malaria.

**Trial registration:**

ClinicalTrials.govNCT04428307, registered June 9, 2020, and NCT04428385, registered June 9, 2020.

**Supplementary Information:**

The online version contains supplementary material available at 10.1186/s13012-020-01077-w.

Contributions to the literature This study presents an innovative approach to improve fever case management in the retail health sector using pragmatic, targeted monetary levers and incentives aimed to motivate decision-making by both retailers and customers based on their individual perspectives during a retail encounter. This diverges from past approaches—which emphasized commodity availability, pricing, and training—and focuses on how customers make decisions about their resources and how businesses embrace diagnostic commodities that have typically been perceived as less marketable than treatments. If successful, this study will provide catalytic evidence for new approaches to improving case management grounded in real-world implementation experience.

## Background

In 2010, the World Health Organization (WHO) released guidance to emphasize the use of parasite-based diagnosis (through malaria rapid diagnostic tests (mRDT) or microscopy) for all suspected cases of malaria [1]. Although there has been a substantial increase in the use of diagnosis in malaria case management during the intervening decade, there is still significant misuse of antimalarial treatments by febrile patients. A modelling exercise estimated that in 2016 a total of 1.1 billion antimalarials (AMs) were used by individuals not infected with malaria [2]. Moreover, between 2015 and 2018, roughly 13% of children in sub-Saharan Africa who had febrile illness and who sought care in private sector pharmacies received a diagnostic test while 41% of them received an antimalarial medicine; despite the majority lacking a confirmatory diagnosis for malaria, greater than 60% of the antimalarials these children received were the WHO recommended treatment, artemisinin-based combination therapies (ACTs) [3].

Global over-consumption of ACTs is largely driven by its increased over-the-counter distribution in private retail outlets as a result of shifts in consumer demand driven by the reduction in retail prices for ACTs due to publicly funded subsidies directed to the private sector [4]. In 2015, 44% of all donor-funded ACTs consumed worldwide were distributed through the private retail health sector where studies have shown that between 65 and 91% of ACTs dispensed for malaria are purchased by people without malaria [5–7]. Overconsumption of ACTs is an unnecessary drain on scarce public health resources and threatens the future sustainability of publicly funded—but private sector-directed—subsidies. To truly transform global malaria case management, new solution-focusing interventions targeting the private sector are required to drive testing uptake, encourage the use of ACTs for positive cases, improve routine data reporting, and better leverage donor resources. Our previous work demonstrated a substantial reduction in the misuse of antimalarials through a successful collaboration between community health workers (CHWs) who offered free community-based diagnostic testing and retail outlets that provided subsidized ACTs to customers with a positive test at the CHW [8].

Following the success of our previous work, we wanted to test implementation of conditional ACT subsidies directly in retail outlets by offering malaria diagnostic testing at the outlets. *Malaria diagnostic testing and conditional subsidies to target ACTs in the retail sector* (TESTsmART) is a randomized experiment of malaria diagnostic testing and conditional subsidies to target ACTs in the retail sector. The study aims to target subsidized ACTs to those who receive a confirmatory diagnosis in private sector retail outlets. To do so, this study proposes a differential price structure that will incentivize providers to offer clients malaria testing and, conditional on a positive test, subsidized ACTs. This approach aims to encourage patients to receive testing and to adhere to results which, when deployed in areas with significant ACT overconsumption, will enhance both sustainability and scalability of mRDT use and ACT targeting. If successful, this intervention could dramatically reduce inappropriate ACT consumption, in alignment with WHO policy that stipulates that all febrile patients be tested before administering antimalarials. Improved targeting will also encourage continued investments in private sector malaria case management from international public health funders because those investments will not result in overconsumption or wastage of ACTs.

This TESTsmART study was originally planned as two linked four-arm 2 × 2 factorial cluster-randomized controlled trials (cRCTs) among registered retail outlets (clusters) conducted separately in two distinct study sites: western Kenya and Lagos, Nigeria. Retail outlets would be allocated evenly across four intervention arms (see “Methods”). As a result of sample size considerations, it was necessary to conduct a three-arm, rather than four-arm, trial in Kenya while keeping the full factorial design in Nigeria. Information about client health and purchasing behavior will be collected through exit interviews with clients emerging from the outlets participating in the study. Data analyses will be conducted separately in each country using a superiority framework for comparison of arms.

## Methods

These methods describe the second of two phases in the TESTsmART study; the first phase [9] aimed to determine the parameters of the incentives intended to be tested in this subsequent study phase. The aim of this second phase of the TESTsmART study is to evaluate the degree to which the incentives, directed at the provider or client, affect the purchasing behavior of all suspected malaria cases seeking treatment with respect to their willingness to undergo diagnostic testing and to purchase appropriate treatments.

### Study setting

**Fig. 1:**
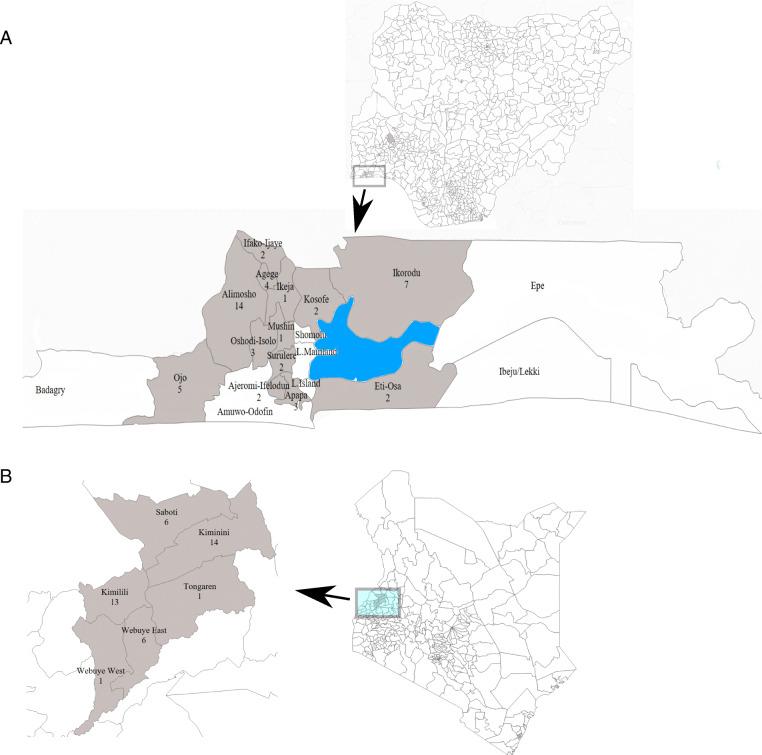
Study settings in western Kenya and Lagos, Nigeria. Shaded areas indicate the areas where the TESTsmART study will be conducted, and the number of clusters in **a** Nigeria and **b** Kenya

The study will be conducted at two sites (Fig. 1): a rural community in a malaria-endemic region of western Kenya where approximately 30% of fevers are due to malaria and malaria rapid testing is not currently available in the private sector, and Lagos, Nigeria, a large urban metropolis where malaria prevalence is around 3% and point-of-care rapid testing through mRDTs has been permitted in private sector retail outlets since 2015 [9, 10]. The trials at each site will be analyzed separately.

In Kenya, the private sector, where 60% of fever cases seek care, is an important source for malaria treatments [8]. Although private retail outlets and chemists are not routinely permitted to conduct mRDTs in Kenya, nationwide surveys show that nearly 71% of ACTs are distributed through the private sector [11]. To ensure adequate regulatory oversight, only retail outlets registered with the Kenya Pharmacy and Poisons Board will be eligible to participate in this study.

In Nigeria, only those outlets registered and licensed by the Pharmaceutical Society of Nigeria are eligible to participate in this study. These outlets, known as Proprietary Patent Medicine Vendors (PPMVs), provide services to febrile patients and regularly stock and sell medicines approved for sale by the Nigerian National Agency for Food and Drug Administration and Control (NAFDAC).

### Interventions

**Fig. 2:**
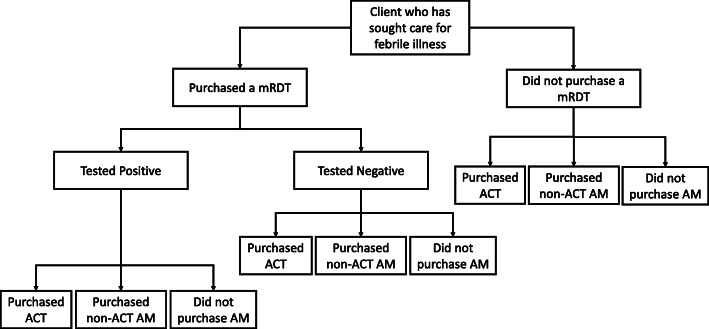
Decision tree of client diagnosis and treatment data collected through exit interviews and by the TESTsmART mobile app

The second phase of the TESTsmART study was planned as two linked four-arm 2 × 2 factorial cluster-randomized trials (Table 1). The unit of randomization (cluster) will be participating private-sector retail outlets at each site. Clients seeking care in participating outlets will be given the option of paying for a mRDT to be conducted at the outlet. The diagnosis and treatment choices made during each transaction between a treatment-seeking client and the outlet provider (Fig. 2) will be captured using a mobile phone app that has been designed specifically for this purpose. The mobile app will also enable us to track and apply provider- and client-directed incentives (see “Data Collection”).

**Table 1:** Description of 2 × 2 factorial design and four study arms

		Client-Directed (CD) Intervention^b^	
		No	Yes
**Provider-directed (PD) intervention**^**a**^	No	Control	Client-directed (CD) intervention only
	Yes	Provider-directed (PD) intervention^c^ only	Combined (PD + CD) interventions

^a^Provider-directed intervention: retail outlets will receive a small incentive to perform a malaria rapid diagnostic test (mRDT) for suspected malaria cases;

^b^Client-directed intervention: clients will receive free artemisinin combination therapy (ACT) if they agree to purchase an mRDT and receive a positive test result;

^c^ The provider-directed (PD) intervention arm will be excluded in western Kenya due to sample size considerations

The four intervention arms are as follows:

1. Control intervention: mRDTs are made available at wholesale price to the retail outlet, and outlet owner/attendant is trained to use the mobile reporting app. mRDTs are offered to clients at a pre-determined price.
2. Provider-directed (PD) intervention: in addition to the interventions implemented in the control outlets, the retail outlet owner in this arm receives a small incentive to perform the mRDT (approximately USD $0.10 in Kenya and USD $0.25 in Nigeria for each mRDT they conduct and report using the mobile app).
3. Client-directed (CD) intervention: in addition to the interventions implemented in the control outlets, clients visiting outlets in this arm receive a free ACT (cost equivalent to 150 Kenyan Shillings (KES) for adults and 60 KES for children in Kenya; 650 Naira for adults and 450 Naira for children in Nigeria) if they purchase an mRDT and receive a positive test result (conditional subsidy).
4. Combined (PD + CD) intervention: in addition to the interventions implemented in the control outlets, retail outlet owners in this arm receive an incentive to test for malaria and clients visiting these outlets receive a free ACT conditional on a positive test result (i.e., this arm is a combination of the PD and CD interventions that are offered in arms 2 and 3 above).

In Nigeria, all four arms will be implemented as originally designed. In Kenya, the provider-directed intervention will be excluded due to sample size considerations that were uncovered through a pilot study, after the original study planning phase, thus leaving us with a three-arm randomized trial (see “Sampling and power calculations” below).

### Study outcomes

A description of each study outcome is shown in Table 2. All client-level outcomes are binary. The primary outcome measure is the proportion of all ACTs that are sold to malaria test-positive clients in each study arm. For our primary outcome, we are interested in evaluating the degree to which the interventions influence the purchasing behavior of all clients seeking treatment; thus, we include in the numerator those clients who were referred from a health facility with a documented positive malaria test result even if no mRDT was subsequently conducted at the retail outlet. For all secondary outcomes, however, we only include those clients who received their mRDT result from the study-enrolled retail outlet to which they presented, regardless of prior testing status. That is, for the secondary outcomes, we are specifically interested in evaluating the degree to which the interventions influenced purchasing behavior among clients who present at the outlet for testing.

**Table 2:** TESTsmART study outcomes

Type	Name	Client sample	Client-level outcome	Arm-level summary	Arm-level formula
Primary	ACT consumption by true malaria cases	All clients who purchased an ACT	1 = positive malaria test^a^ and purchases ACT 0 = otherwise	Proportion of ACTs that are sold to malaria test-positive^a^ clients	$\frac{\#\mathrm{clients}\ \mathrm{who}\ \mathrm{purchased}\ \mathrm{ACT}\ \mathrm{and}\ \mathrm{tested}\ \mathrm{positive}}{\#\mathrm{clients}\ \mathrm{who}\ \mathrm{purchased}\ \mathrm{ACT}}$
Secondary	Use of malaria rapid diagnostic test	All suspected malaria cases^b^	1 = tested with mRDT 0 = otherwise	Proportion of suspected malaria cases that receives a malaria test	$\frac{\#\mathrm{suspected}\ \mathrm{malaria}\ \mathrm{cases}\ \mathrm{tested}\ \mathrm{with}\ \mathrm{mRDT}}{\#\mathrm{suspected}\ \mathrm{malaria}\ \mathrm{cases}}$
	Adherence to mRDT result	All clients who were tested with mRDT	1 = positive malaria test and purchases ACT OR negative malaria test and did not purchase any AM 0 = otherwise	Proportion of malaria tested clients whose treatment adhered to test results	$$ {\displaystyle \begin{array}{c}\#\mathrm{clients}\ \mathrm{that}\ \mathrm{tested}\ \mathrm{positive}\ \mathrm{with}\ \mathrm{mRDT}\ \mathrm{and}\ \mathrm{purchase}\mathrm{d}\ \mathrm{ACT}+\\ {}\frac{\#\mathrm{clients}\ \mathrm{that}\ \mathrm{tested}\ \mathrm{negative}\ \mathrm{with}\ \mathrm{mRDT}\ \mathrm{and}\ \mathrm{d}\mathrm{id}\ \mathrm{not}\ \mathrm{purchase}\ \mathrm{AM}}{\#\mathrm{clients}\ \mathrm{tested}\ \mathrm{with}\ \mathrm{mRDT}}\end{array}} $$
	Appropriate case mgmt	All suspected malaria cases^b^	1 = positive malaria test and purchases ACT OR negative malaria test and did not purchase any AM 0 = otherwise	Proportion of suspected malaria cases that are managed appropriately	$$ {\displaystyle \begin{array}{c}\#\mathrm{clients}\ \mathrm{that}\ \mathrm{tested}\ \mathrm{positive}\ \mathrm{with}\ \mathrm{mRDT}\ \mathrm{and}\ \mathrm{purchase}\mathrm{d}\ \mathrm{ACT}+\\ {}\frac{\#\mathrm{clients}\ \mathrm{that}\ \mathrm{tested}\ \mathrm{negative}\ \mathrm{with}\ \mathrm{mRDT}\ \mathrm{and}\ \mathrm{d}\mathrm{id}\ \mathrm{not}\ \mathrm{purchase}\ \mathrm{AM}}{\#\mathrm{suspected}\ \mathrm{malaria}\ \mathrm{cases}}\end{array}} $$
	ACT use among the untested	All untested clients	1 = purchased ACT 0 = otherwise	Proportion of untested clients taking ACT	$\frac{\#\mathrm{untested}\ \mathrm{clients}\ \mathrm{who}\ \mathrm{purchased}\ \mathrm{ACT}\ }{\#\mathrm{untested}\ \mathrm{clients}\ }$

^a^mRDT+ based on RDT conducted at retail outlet or positive by test conducted outside of the retail outlet when documentation is provided

^b^A “suspected malaria case” is any client who was tested with an mRDT or was untested but purchased any antimalarial (AM)

The major secondary outcome is the proportion of suspected malaria cases that are tested, where we define a suspected malaria case as any client who was tested with a mRDT at the outlet or who was untested but purchased any antimalarial at the outlet. This outcome will allow us to determine whether the conditional subsidy can drive demand for testing. Other secondary outcomes include adherence to mRDT results (the proportion of clients who properly adhered to their mRDT result out of all clients receiving an mRDT) and appropriate case management (the proportion of clients who properly followed their mRDT result, with respect to follow-on treatment options, out of all suspected malaria cases). We defined adherence to the mRDT result as purchasing an ACT if they tested positive and not purchasing any antimalarial (AM) if they tested negative, and we define a client as a suspected malaria case if they were tested with a mRDT or they were untested but purchased any AM. Finally, we will also calculate the proportion of clients taking ACTs without a diagnostic test.

Study outcomes will be collected through exit interviews with clients who sought care for a febrile illness at each of the enrolled retail outlets. All outcomes will be derived based on data collected during the 15-month collection window.

### Sampling and power calculations

The primary comparison of interest is the effect on our primary outcome of offering a combination of provider-directed and client-directed interventions relative to the control arm (i.e., PD + CD arm vs. control arm). In order to evaluate whether the provider-directed or client-directed interventions have a synergistic effect on the outcome, two secondary comparisons are of interest: (1) PD vs. PD + CD arms and (2) CD vs. PD + CD arms, where only the latter comparison can be estimated in Kenya due to the three-arm trial design. We powered the study to analyze significant changes in the aforementioned comparisons (Table 3). We expect that the client-directed intervention (CD) will have a somewhat larger effect, and that the largest effect will come from combining the two interventions (PD + CD) (i.e., we assume a statistical interaction).

**Table 3:** Estimated power in Nigeria and Kenya

	Estimated power in Nigeria		Estimated power in Kenya	
Primary outcome comparison	4-arm design with a total of 48 clusters (alpha = 0.05/3 = 0.0167)		3-arm design with a total of 40 clusters (alpha = 0.05/2 = 0.025)	
	Expected effect size	Power	Expected effect size	Power
Combined incentives (PD + CD) vs. control	68% (PD + CD) − 21% (control) = **47 percentage points**	100%	23% (PD + CD) − 7% (control) = **16 percentage points**	99.1%
Combined incentives (PD + CD) vs. provider-incentives (PD)	68% (PD + CD) − 24% (PD) = **44 percentage points**	100%	NA	NA
Combined incentives (PD + CD) vs. Client-incentives (CD)	68% (PD + CD) − 38% (CD) = **30 percentage points**	98.8%	23% (PD + CD) − 12% (CD) = **11 percentage points**	80.2%

Expected effect size, change in percentage of ACTs taken by clients with a positive test. Since our outcome is a composite measure of testing rates and adherence to the test result, our sample size calculations accounted for the fact that not everyone who we interview will have taken an ACT. For Nigeria, power was calculated based on 12 clusters per arm for a total of 48 clusters. For Kenya, power was calculated based on 13 clusters per arm. In practice an additional cluster will be available in the control arm in Kenya, for a total of 14 clusters in this arm, for a total of 40 clusters

Sample sizes were calculated with original hypothesized effect sizes and re-evaluated with pilot data separately for Kenya and Nigeria, using the formula from Moulton and Hayes for comparing two proportions under a cluster-randomized trial design [12]. We estimated the intra-class correlation coefficients (ICCs) for the primary outcome to be 0.009 in Kenya and 0.01 in Nigeria. We determined the minimum sample sizes required for 90% power to detect the original hypothesized effect sizes for each of the three main comparisons of interest and chose the largest sample size required. Hypothesized effect sizes and power were re-evaluated with pilot data for Kenya and Nigeria (Table 3). To ensure overall two-tailed type I error (alpha) control at 0.05 in each country, the conservative Bonferroni correction was used to fix the alpha level for each comparison at 0.05/3 = 0.0167 in Nigeria and 0.05/2 = 0.025 in Kenya [13]. See Supplemental File 1 for a detailed description of the sample size calculations, including derivation of the ICC estimates, effect sizes, and the different assumptions between countries.

Note that the difference in effect sizes between Nigeria and Kenya is due to different assumptions about client behavior in each country, informed by our understanding of the local health context. Since our outcome is a composite, different assumptions about the pathway to client ACT use result in different effect sizes. In general, we expect a higher proportion of clients receiving an mRDT and a lower proportion of negative/untested clients taking ACT in Nigeria compared to Kenya.

Pilot data in Kenya indicated slightly lower test positivity than expected; calculations based on this pilot data indicated we might only reach 59.5% power for the comparison of the combined PD + CD arm and the CD arm. By collapsing from 4 arms to 3 and increasing the number of clusters per arm (from 10 to 13), we expect to achieve 80.2% power for this comparison. We decided to keep the CD arm, rather than PD arm, since private outlets in Kenya are permitted to stock and sell ACTs but are not (yet) allowed to conduct mRDTs outside of research settings.

In Kenya, our sample will include 40 retail outlets with 14 outlets assigned to the control arm and 13 outlets assigned to each of the CD and PD + CD arms while in Nigeria our sample will consist of 48 retail outlets equally assigned to each of the four study arms. Within each of these outlets, we will have 170 exit interviews, resulting in a total sample size of 6800 in Kenya (170 × 40) and 8160 in Nigeria (170 × 48).

### Enrollment of retail outlets and their assignment into study arms

In each country, among those private registered retail outlets and PPMVs that expressed interest in participating in the study, eligible private outlets were identified according to set criteria (Table 4).

**Table 4:** Eligibility criteria for retail outlet enrollment in the TESTsmART study

Study eligibility criteria^a^	Response required for study eligibility
Does the outlet routinely stock and sell ACTs?	Yes
Are they willing to acquire mRDTs and use in diagnosing malaria for patients?	Yes
Are they willing to use a phone/app to collect/report data and receive subsidy?	Yes
Are they willing to allow a data collector to conduct patient exit interviews for several days each month at the outlet/PPMV?	Yes
Is the outlet license/registration up to date?	Yes

^a^In Nigeria, PPMVs were also excluded if they had challenges with network connectivity at the outlet, if they were participating in other NGO projects, or if they had any agreements with drug/diagnostic marketers

**Fig. 3:**
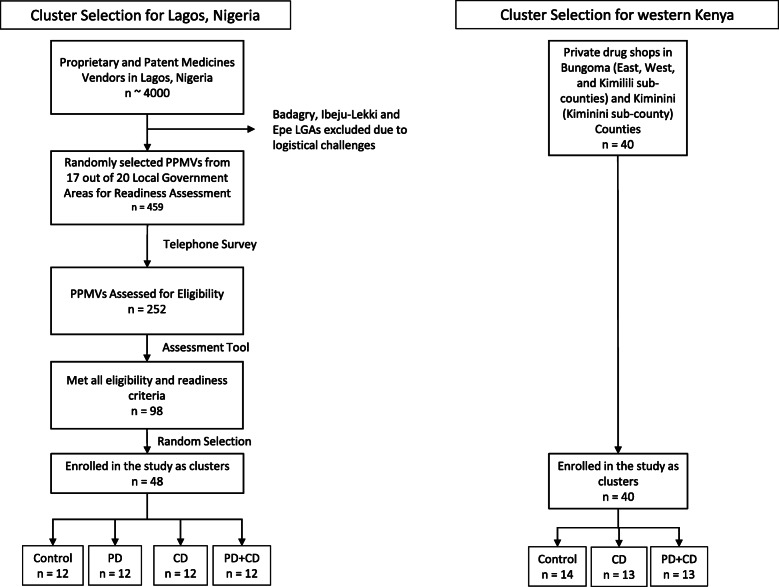
Selection of retail outlets for enrollment in the TESTsmART study

In Kenya, a full sampling frame of eligible retail outlets was generated for each county. Outlets were randomly selected for enrollment from this sampling frame; if an outlet declined enrollment, they were replaced with one of the remaining outlets in that county. In Nigeria, a complete listing of PPMVs was stratified across three geographical regions of the city and outlets were enrolled from a random subset proportional to the size of the strata (Fig. 1). Following enrollment, retail outlets (Fig. 3) were randomized to arms separately within each country by the study statistician. In Kenya, a uniform random number between 0 and 1 was generated for all 40 outlets, then sorted and split into 3 groups using the quantiles of the distribution stratified by county. Those groups were labeled A, B, and C, and those labels were randomized so that they were allocated to one of the three trial arms. Given 40 outlets cannot be equally allocated to 3 arms, the extra outlet was assigned to the control arm. In addition, to avoid potential contamination of treatment effects, randomization was constrained such that any outlets in close proximity (< 0.5 km) were assigned to the same arm. In Nigeria, we used the same randomization process, but with allocation to 4 arms.

All outlets have access to mRDTs at a wholesale price, and the retail price of the mRDTs will be consistent across all arms within each country setting. The four-arm study design in Nigeria and three-arm design in Kenya will allow us to measure the effect of the combined PD + CD intervention relative to no intervention or either intervention (PD or CD) alone.

### Data collection

Due to the nature of the interventions, it is not possible to blind participants and the implementation team to the study arm allocated to each outlet. Data collectors will be blinded throughout collection, and study statisticians will be blinded during the analysis phase. Data collection will proceed via two methods: the health care providers’ use of the TESTsmART mobile app for real-time monitoring, and exit interviews conducted at each outlet for measuring outcomes. Data for main study outcomes will be collected by exit interviews with clients in order to avoid bias that may arise by relying on provider-reported data. Exit interviews and provider reporting data will be compared to assess agreement between both sources.

### Data collection via the TESTsmART mobile app

Private providers will report routine data using the TESTsmART mobile application. The data collected through the TESTsmART app will be synced to a central server to allow for remote monitoring. All outlet attendants within each enrolled outlet will be trained to use the mobile app which reports on volume of clients, number of ACTs or other antimalarials sold, and number of mRDTs sold. Data reported through the app will primarily be used to track mRDT and ACT sales in real-time and will be regularly reviewed to tabulate and track the outlet-specific test positivity rate, volume of RDTs used, and visualization of a random sampling of uploaded mRDT photos. This routine monitoring will detect potential problems (e.g., providers who have unusually high- or low-test positivity rate or errors in mRDT interpretation). Problems detected through routine monitoring will trigger supportive supervision and/or additional on-the-job training to ensure compliance and quality of diagnosis.

The app is designed to enhance future scalability of the intervention. Via the app “dashboard,” the study team will review the mRDTs, test results, and ACT sales to calculate the payment to each outlet based on their arm assignment. This will be done weekly and implemented through mobile money platforms in each country.

### Data collection via cluster-based exit interviews

Interviews will be conducted with clients departing from participating retail outlets (study clusters) on random days of the week. All clients exiting the outlet that day are eligible to be screened, and field researchers are instructed to make no pre-judgements about clients but rather approach the next available client exiting the outlet. Interviewees must meet specific eligibility criteria to proceed with the exit interview (Table 5). We aim to complete exit interviews with one hundred and seventy clients enrolled per outlet over the course of the 15-month intervention period. The study team will conduct a verbal consent process for participants who meet the inclusion/exclusion criteria, prior to participation in the exit interview outside the outlets.

**Table 5:** Eligibility criteria for exit interview subjects

Inclusion criteria	Exclusion criteria
Have fever, or History of fever in the last 48 h, or Suspects that they may have malaria	Have severe illness requiring immediate referral
Must be present at point of recruitment	Have taken an antimalarial in the last 7 days, including for the current illness
Be older than 1 year of age	Be < 18 years of age without a parent or legal guardian present
	Unable to consent

### Data management

Data for participant exit interviews will be collected electronically via tablet. The data will be encrypted and password protected. In Kenya, tablets are locked in a secure cabinet nightly, and data are removed several times per week. In Nigeria, data will be transferred from the tablet to a secure, password-protected computer once per week. The primary tool for developing the data collection forms will be REDCap™ hosted at Duke University, which is a free, secure, web-based application for data capture [14, 15]. REDCap™ provides built-in data validation (e.g., data types, range checks) for quality assurance over data entry and an electronic audit trail that permanently tracks and logs every access of data, tools, or reports within the database. Individuals will be assigned a unique study ID, and only anonymous data will be collected in this study. A data-monitoring committee was deemed unnecessary by the funder because this study is minimal risk and tests an intervention designed to influence behavior and decision-making. Furthermore, we are not collecting any protected health information, and only anonymous data will be used in this study. On completion of the trial and publication of trial findings, the final trial dataset may be available to investigators if requested from the authors.

### Data analysis

We will analyze client-level outcomes by fitting a modified Poisson regression model [16, 17] with log link to estimate risk ratios (RRs) and identity link to estimate risk differences. Such an approach assumes a Poisson distribution for the binary outcome and then “fixes” the estimated standard errors to correct for model misspecification.

To account for clustering by outlet, we will use a generalized estimating equations (GEE) [18, 19] approach with exchangeable working covariance matrix and robust standard errors (to correct for model misspecification due to specifying a Poisson distribution). The outcome will be regressed on three binary indicators for each of control, PD, and CD, with treatment arm PD + CD (the combined interventions) serving as the reference group noting that the indicator for the PD will be excluded in the three-arm Kenya study. The model will also include fixed effects for the stratification variables and a vector of potential confounder variables (e.g., age, gender, education, or other socioeconomic indicators) to account for possible imbalances between study arms. All analyses will be based on the intention-to-treat principle whereby all clients will be included in the analysis irrespective of whether they complied with the intervention in the outlet at which they sought care (e.g., even if they did not use the ACT subsidy if they tested positive in an outlet in CD and PD + CD that received the client-directed intervention). Since we do not have longitudinal follow-up, we will not need to account for missing data due to attrition of clients. Patterns of client non-response will be described and compared by outlet and between arms. Given our prior experience in these regions, client non-response is anticipated to be minimal and comparable between arms.

Given that the literature indicates that when there are fewer than 40 clusters in a cRCT, small sample correction methods should be used to ensure that standard error estimates are correctly estimated when using GEE to analyze binary outcomes, and given that the size of the cRCTs in each country are close to this cutoff, we plan to adopt the use of the Kauerman-Carroll correction to avoid any possible problems [20, 21]. We will compare secondary outcomes using the same modeling approach.

### Commodity supply to study participants

To ensure availability of mRDTs, the research team will set up a supply chain for participating retail outlets to access affordable and quality-assured mRDTs. The mRDT price that retail outlets will pay is set by study team. In both countries, retail outlets will be trained in reorder procedures during the study training at the onset of the study. All outlets will receive an initial stock of two kits or 50 mRDTs, free of charge. Other auxiliary items, like gloves, a sharp container and waste bags will also be provided during the trainings. The sales price of the mRDT set by the study team approximately tracks with the median retail prices of mRDTs found in the public sector in Kenya or in other community retail outlets in Nigeria.

All retail outlets participating in the study will obtain ACTs through existing distribution channels and suppliers.

### Study timeline

Retail outlets participating in the study have been recruited, and training of staff on the use of the TESTsmART app commenced in June 2020. The study began assignment of retail outlets into study arms in October 2020, with retail outlets commencing transactions and data collection under the intervention structure provided for their respective study arm. The study will be implemented for a 3 month burn-in period before exit interview data collection begins in January 2021 (Kenya) and February 2021 (Nigeria). Data collection will continue through April 2022 (Kenya) and May 2022 (Nigeria), with data analysis conducted thereafter.

Trial results will be published as soon as possible upon completion of the study and will also be available on Clinical Trials.gov one year after the end of enrollment. We will post the trial description, data collection forms, and data structure on our institutional website as soon as the primary manuscript is accepted for publication. The requestor will be able to contact the principal investigator at a link on the website to request data. De-identified datasets containing participant-level data will be made available to the user with the following stipulations:

- The data will be used for research purposes and not to attempt to identify individual subjects
- The data must be stored securely and destroyed after analyses are complete
- The authors of any manuscript resulting from this data must acknowledge the source of the data upon which their manuscript is based.

## Discussion

Here, we describe a real-world implementation study that seeks to incorporate differential pricing structures into business transactions to guide drug consumption decisions. The outcomes will be measured among real customers seeking care for febrile illness in the retail health sector. This is a departure from other approaches to private sector engagement which heavily rely on training and provision of commodities and measure outcomes based on retailer behavior. As a result of our study design and intervention strategy, the evidence generated will be directly applicable to interventional scale up without the need for further adaptation.

This study protocol describes two linked 2 × 2 factorial cluster-randomized controlled trials examining an intervention for improving the rational use of ACTs for the treatment of malaria by engaging the retail health sector. This study aims to intervene at the retail health sector level to test whether provider incentives to offer mRDTs and conditional subsidies for clients with a positive diagnostic test can help improve the rational use of ACTs in Kenya and Nigeria. Our study design will allow us to measure the individual as well as combined effects of the provider and client-directed incentives.

If our study demonstrates that appropriate malaria case management can be improved by placing a greater emphasis on offering malaria diagnostic testing in the retail health sector where most clients purchase ACTs, it could reduce overconsumption of ACTs and enhance targeting of publicly-funded ACT subsidies to those who need them. It may also extend the useful therapeutic life of first-line malaria drugs. Although there may be challenges in scale-up of RDT testing, linking testing and treatment may increase demand for affordable diagnostic testing services. This presents an opportunity for the retail health sector where many clients are choosing to purchase treatments. Increasing access to diagnostic testing in the retail health sector will require an investment in training, but many local governments have several models for successfully training alternative cadres of healthcare professionals in order to expand testing services that can be adapted to retail outlet attendants. Improving testing before treatment of suspected malaria cases in the private sector could ultimately harmonize public health policy and on the ground practice.

## Supplementary Information

**Additional file 1.** Sample Size Calculations. Additional information and data tables describing the sample size and power calculations for this study.

## Data Availability

Data sharing is not applicable to this article as no datasets were generated or analyzed during the current study.
