# Correction to: Incentivizing appropriate malaria case management in the private sector: a study protocol for two linked cluster randomized controlled trials to evaluate provider- and client-focused interventions in western Kenya and Lagos, Nigeria

**DOI:** 10.1186/s13012-021-01093-4

**Published:** 2021-03-16

**Authors:** Aaron M. Woolsey, Ryan A. Simmons, Meley Woldeghebriel, Yunji Zhou, Oluwatosin Ogunsola, Sarah Laing, Tayo Olaleye, Joseph Kipkoech, Bomar Mendez Rojas, Indrani Saran, Mercy Odhiambo, Josephine Malinga, George Ambani, Emmah Kimachas, Chizoba Fashanu, Owens Wiwa, Diana Menya, Jeremiah Laktabai, Theodoor Visser, Elizabeth L. Turner, Wendy Prudhomme O’Meara

**Affiliations:** 1 Clinton Health Access Initiative, Boston, MA USA; 2 Duke Global Health Institute, Duke University, Durham, NC USA; 3 Department of Biostatistics and Bioinformatics, Duke University, Durham, NC USA; 4 Clinton Health Access Initiative, Lagos, Nigeria; 5 Academic Model Providing Access to Healthcare, Moi University, Eldoret, Kenya; 6 School of Social Work, Boston College, Boston, MA USA; 7 Clinton Health Access Initiative, Abuja, Nigeria; 8 College of Health Sciences, Moi University School of Public Health, Eldoret, Kenya; 9 College of Health Sciences, Moi University School of Medicine, Eldoret, Kenya; 10 Department of Medicine, Duke University, Durham, NC USA

**Correction to: Implementation Sci (2021) 16:14**

**https://doi.org/10.1186/s13012-020-01077-w**

Following publication of the original article [1], it was reported that the incorrect version of Additional file 1 was uploaded.

The correct files for Additional file 1 are included in this Correction article and the original article has been updated.

## Supplementary Information

**Additional file 1.**
