# Correction: Incentivizing appropriate malaria case management in the private sector: a study protocol for two linked cluster randomized controlled trials to evaluate provider- and client-focused interventions in western Kenya and Lagos, Nigeria

**DOI:** 10.1186/s13012-022-01233-4

**Published:** 2022-09-15

**Authors:** Aaron M. Woolsey, Ryan A. Simmons, Meley Woldeghebriel, Yunji Zhou, Oluwatosin Ogunsola, Sarah Laing, Tayo Olaleye, Joseph Kipkoech, Bomar Mendez Rojas, Indrani Saran, Mercy Odhiambo, Josephine Malinga, George Ambani, Emmah Kimachas, Chizoba Fashanu, Owens Wiwa, Diana Menya, Jeremiah Laktabai, Theodoor Visser, Elizabeth L. Turner, Wendy Prudhomme O’Meara

**Affiliations:** 1 Clinton Health Access Initiative, Boston, Massachusetts USA; 2 Duke Global Health Institute, Duke University, Durham, North Carolina USA; 3 Department of Biostatistics and Bioinformatics, Duke University, Durham, North Carolina USA; 4 Clinton Health Access Initiative, Lagos, Nigeria; 5 Academic Model Providing Access to Healthcare, Moi University, Eldoret, Kenya; 6 School of Social Work, Boston College, Boston, Massachusetts USA; 7 Clinton Health Access Initiative, Abuja, Nigeria; 8 Moi University School of Public Health, College of Health Sciences, Eldoret, Kenya; 9 Moi University School of Medicine, College of Health Sciences, Eldoret, Kenya; 10 Department of Medicine, Duke University Durham, North Carolina, USA

**Correction: Implement Sci 16, 14 (2021)**

**https://doi.org/10.1186/s13012-020-01077-w**

Following publication of the original article [1] the authors reported a correction to their published study protocol.

The purpose of this correction is to update the design of one of the two linked cluster randomized trials (CRTs) in our study protocol entitled “*Incentivizing appropriate malaria case management in the private sector: a study protocol for two linked cluster randomized controlled trials to evaluate provider- and client-focused interventions in western Kenya and Lagos, Nigeria”* [1] and to provide justification for a lower target sample size in the other trial. This study included a CRT enrolling clusters (retail outlets) into four treatment arms in Nigeria using estimated sample sizes based on malaria prevalence and testing rates in the published literature. However, after an initial period of seven months of data collection, we observed considerable differences between the initial study assumptions and actual observations (we note that we only examined data combined across the four study arms and did not examine the data stratified by treatment arm so as to avoid unblinding the study team to treatment arm outcome data). In particular, we observed lower than expected treatment seeking for malaria-like illness (only 27% of patients approached in retail shop exit interviews met the study inclusion criteria across all four treatment arms), and lower testing rates among those who were eligible for study inclusion (20% testing rate across all four treatment arms in actual observations, compared to our original assumption of 65% testing rate across all four treatment arms). Differences on other key assumptions were also observed (Table A1). The combination of these differences has considerably reduced the power to detect differences in the primary outcome of the study, namely in ACT consumption by true malaria cases (Table A2: comparison of power based on observations from the first seven months of data collection).

**Table A1: Updated assumptions about testing/ACT uptake and primary outcome in Nigeria based on blinded data from an initial period of seven months of data collection**

**Table:** 

**ASSUMPTIONS ABOUT TESTING/ACT UPTAKE for Nigeria CRT**				
	**Control**	**PD** **(provider directed intervention)**	**CD** **(client directed intervention)**	**PD+CD** **(combined interventions)**
***Fevers tested***	9%	14%	14%	39%
***Tested fevers that are positive***	18%	18%	18%	18%
***Positive fevers that take ACT***	48%	48%	78%	78%
***Negative fevers that take ACT***	35%	35%	27%	27%
***Untested fevers that take ACT***	42%	42%	42%	42%
***Positive fevers that take non-ACT Am***	36%	36%	14%	14%
***Negative fevers that take non-ACT Am***	15%	15%	15%	15%
***Untested fevers that take non-ACT Am***	26%	26%	26%	26%
**PRIMARY OUTCOME**				
	**Control**	**PD** **(provider directed intervention)**	**CD** **(client directed intervention)**	**PD+CD** **(combined interventions)**
**ACT consumption by true malaria cases ( i.e.** ***% of ACTs used by positives)***	2%	3%	5%	14%

Notes: Given that we examined actual observed data combined across all four study arms, for which we observed an overall testing rate of 20% rather than the 65% expected based on published literature, the updated proportions with the primary outcome in each arm of the 4-arm design were estimated based on shifting testing rates down by 45 percentage points in each of the four study arms, as well as adjustment to some of the other key assumptions

*ACT* Artemisinin Combination Therapies, *Am* Antimalarial medicine(s)

To address the anticipated loss of power for the original study design, we have taken the following steps:

1. We have revised the study design for the Nigeria CRT, changing it from a four-arm study to a two-arm study, proceeding with only the control arm and the combined interventions arms. This new CRT design has been updated on ClinicalTrials.gov (ID: NCT04428385). The new study design will result in adequate power to test for a meaningful intervention effect on the primary outcome when comparing the combined intervention arm vs. the control arm (Table A2: Two arm sample size and power calculation). To be consistent with the original power calculation, we used the formulae from Hayes and Moulton [2] for the derivation of intraclass correlation coefficient (ICC) and for comparing two proportions under a cluster-randomized trial design (see Supplemental File 1, previously published). In order to account for varying cluster sizes, we modified the Hayes and Moulton formula for comparing two proportions [3] by replacing the cluster size (*m*) in with *m*/(1 + *CV*^2^), where CV is the coefficient of variation of cluster size. These changes are reflected in the Clinicaltrials.gov NCT04428385 entry covering the Nigeria CRT.

**Table A2. Assessment of power for the primary outcome of "ACT consumption by true malaria cases” based on initial data collection for the Nigeria CRT**

**Table:** 

	Power based on 4-arm design		Power based on 2-arm design	
Key Assumptions	12 clusters per arm (alpha=0.05/3 = 0.0167); m = 32; ICC = 0.037; CV = 0		24 clusters per arm (alpha=0.05); m = 32; ICC = 0.037; CV = 0.72	
Primary Outcome Comparison	Expected Effect Size	Power	Expected Effect Size	Power
Combined Interventions (PD+CD) vs. Control Arm	14% (PD+CD) – 2% (Control) = **12 percentage points**	54.4%	14% (PD+CD) – 2% (Control) = **12 percentage points**	91.4%
Combined Interventions (PD+CD) vs. Provider Directed Intervention (PD)	14% (PD+CD) – 3% (PD) = **11 percentage points**	43.5%	NA	NA
Combined Interventions (PD+CD) vs. Client Directed Intervention (CD)	14% (PD+CD) – 5% (CD) = **9 percentage points**	26.6%	NA	NA

Notes: m, expected median cluster size by the end of the study; ICC, intraclass correlation coefficient; CV, coefficient of variation of cluster size

2) We have re-allocated the participating retail shops from four arms into the revised 2-arm study design (control arm and combined intervention arm). The intervention arm includes both the client-directed and provider-directed incentives. We recognized that we will be unable to estimate the effects of the provider-directed and client-directed incentives individually in this new design. Instead, we will estimate their combined effect on the outcome.
3) In the process of re-allocating the participating shops/clusters, the control and client-directed incentive arms of the study were collapsed into a new control arm while the provider-directed incentive and combined incentive arms of the prior study design were collapsed into a new combined-intervention arm.
4) Furthermore, we dropped eight participating shops from which we had been unable to collect sufficient or any data over a five-month period. Reasons for lack of data collection included: shops that had closed permanently or opened only very erratically, very low client volumes, shop opening hours during evenings (when it was not safe to conduct exit interviews) or weekends only. All eight shops were in the new combined-intervention arm. Based on discussions with the owners of these eight retail outlets, it did not appear that the problems leading to discontinuation of these shops was related to the intervention or arm assignment and therefore is believed to be random with respect to the study design and independent of the intervention components. To maintain both the geographical distribution of study-enrolled shops and the average data collection rates per arm, we then randomly switched four of shops from the new control arm into the combined intervention arm. The random selection of the four shops from control arm was stratified by geographical location to match the original design and was restricted to those with low shop volumes (# of exit interviews as of September 2021: lower than eighteen). This criterion was chosen because we observed a balance between the retail outlets in the combined intervention arm and the control arm in terms of the number of outlets with larger client volume but that after dropping the eight formerly poorly-participating shops, we observed a substantial imbalance between the two arms in the number of low-volume shops.
5) We then added eight shops to the study that met the inclusion criteria and matched feasibility of data collection with the shops that remained enrolled (verified through visits to the outlets). The new PPMVs were randomly assigned to each of the two arms, stratified by geographical area.
6) Data collection has been extended for the Nigeria CRT for an additional year to accommodate these changes. The primary analysis will only include data collected after the design change, with sensitivity analysis using relevant information collected before the design change.
7) Full data analysis plan is available on clinical trials.gov. Analysis will be for a two-arm comparison and no longer based on data from the four arms of a 2x2 factorial design.
8) The outcome measures have not changed but to reiterate, we will no longer be able to estimate the independent effects of the provider-directed or client-directed interventions in Nigeria.
9) Power for the Kenya CRT was assessed with actual observations in the same way. We observed lower than expected treatment seeking for malaria-like illness and other slight differences on key assumptions (Table A3). However, given the updated key assumptions and an expected median cluster size of 79 instead of the original target of 170, we will still be able to achieve 86.0% and 99.8% power for the PD+CD vs. CD and PD+CD vs. Control comparisons respectively (Table A4). Therefore, we confirmed that a lower sample size is acceptable and a major design change is not necessary for the Kenya CRT.

**Table A3: Updated assumptions about testing/ACT uptake and primary outcome in Kenya based on blinded data from an initial period of ten months of data collection**

**Table:** 

**ASSUMPTIONS ABOUT TESTING/ACT UPTAKE FOR KENYA CRT**			
	**Control**	**CD** **(client directed intervention)**	**PD+CD** **(combined interventions)**
***Fevers tested***	23%	28%	53%
***Tested fevers that are positive***	47%	47%	47%
***Positive fevers that take ACT***	49%	74%	74%
***Negative fevers that take ACT***	39%	29%	29%
***Untested fevers that take ACT***	73%	73%	73%
***Positive fevers that take non-ACT Am***	29%	4%	4%
***Negative fevers that take non-ACT Am***	3%	3%	3%
***Untested fevers that take non-ACT Am***	8%	8%	8%
**PRIMARY OUTCOME**			
	**Control**	**CD** **(client directed intervention)**	**PD+CD** **(combined interventions)**
**ACT consumption by true malaria cases ( i.e.** ***% of ACTs used by positives)***	8%	14.6%	30%

Notes: Given that we examined actual observed data combined across all three study arms, the updated proportions with the primary outcome in each arm of the 3-arm design were estimated based on shifting the key assumptions by the same magnitude (i.e., the difference between the aggregated proportion across all arms and the original assumption aggregated across arms) in each of the three study arms

*ACT* Artemisinin Combination Therapies, *Am* Antimalarial medicine(s)

**Table A4: Estimated power for primary outcome based on assumptions updated with aggregated exit interview data from first ten months of implementation of the three-arm trial in Kenya**

**Table:** 

	Power based on three-arm design	
Key Assumptions	13 clusters per arm (alpha=0.05/2=0.025); m = 79; ICC = 0.012; CV = 0.47	
Primary Outcome Comparison	Expected Effect Size	Power
Combined Interventions (PD+CD) vs. Control Arm	30% (PD+CD) – 8% (Control) = **22 percentage points**	99.8%
Combined Interventions (PD+CD) vs. Client Directed Intervention (CD)	30% (PD+CD) – 14.6% (CD) = **15.4 percentage points**	86.0%

Notes: m, expected median cluster size by the end of the study; ICC, intraclass correlation coefficient; CV, coefficient of variation of cluster size

10) Updated grant award number: R01 AI141444
