# Supplementary material for: Incentivizing appropriate malaria case management in the private sector: a study protocol for two linked cluster randomized controlled trials to evaluate provider- and client-focused interventions in western Kenya and Lagos, Nigeria
Source: Implement Sci. 2021 Jan 20;16:14. doi: 10.1186/s13012-020-01077-w (PMC7816435; doi:10.1186/s13012-020-01077-w)
Supplement: Supplementary file 1 — Additional file 1. Sample Size Calculations. Additional information and data tables describing the sample size and power calculations for this study. [file 13012_2020_1077_MOESM1_ESM.pdf]

Incentivizing appropriate malaria case management in the private sector: a study protocol for two linked cluster randomized controlled trials to evaluate provider- and client-focused interventions in western Kenya and Lagos, Nigeria

Aaron M Woolsey[1,*], Ryan A Simmons[2,3], Meley Woldeghebriel[1], Yunji Zhou[2,3], Oluwatosin Ogunsola[4], Sarah Laing[2], Tayo Olaleye[4], Joseph Kipkoech[5], Bomar Mendez Rojas[1], Indrani Saran[6], Mercy Odhiambo[1], Josephine Malinga[2], George Ambani[5], Emmah Kimachas[5], Chizoba Fashanu[7], Owens Wiwa[7], Diana Menya[8], Jeremiah Laktabai[5,9], Theodoor Visser[1], Elizabeth L Turner[2,3], Wendy Prudhomme O'Meara[2,8, 10,†]

Sample Size Calculation

Sample sizes were first calculated with the original hypothesized effect sizes that were included in the grant submission. These calculations were then re-evaluated using pilot data obtained during the first phase of the TESTsmART study. In all cases, a standard sample size formula was used, namely that from Moulton and Hayes for comparing two proportions under a cluster-randomized trial (CRT) design, and including a penalty of an additional two clusters per arm because the standard sample size formula is for "large samples" but the two linked trials outlined in the protocol include between 12 and 14 clusters per arm(see below) [1]. We estimated the intra-class correlation coefficients (ICCs) for the primary outcome to be 0.009 in Kenya, and 0.01 in Nigeria (Table S1). We determined the minimum sample sizes required for 90% power to detect the original hypothesized effect sizes (Tables S2-S3) for each of the three main comparisons of interest and chose the largest sample size required (Table S4). Hypothesized effect sizes and power were re-evaluated with pilot data for Kenya (Tables S5-S6) and Nigeria (Tables S7-S8). To ensure overall two-tailed Type I error (alpha) control at 0.05 in each country, the conservative Bonferroni correction was used to fix the alpha level for each comparison at 0.05/3=0.0167 in Nigeria and in the original calculations for Kenya and, later using 0.05/2=0.025 in Kenya, based on a modification to a three-arm CRT [2].

Based on the power calculation, our sample in Kenya will include 40 retail outlets with 14 outlets assigned to the control arm and 13 outlets assigned to each of CD and PD+CD, while in Nigeria our sample will consist of 48 retail outlets equally assigned to each of the four arms. Within each of these outlets, we will have 170 exit interviews, resulting in a total sample size of 6800 in Kenya (170 X 40) and 8160 in Nigeria (170 X 48).

Standard formulae for the comparison of two proportions for cluster-randomized trials

In a CRT, with m individuals sampled from each cluster, c, the required number of clusters per arm, is given by equation (8.6) in Hayes and Moulton (2009) [1]:

$$c = 1 + \left(z_{\alpha/2} + z_{\beta}\right)^2 \frac{\pi_0(1-\pi_0)/m + \pi_1(1-\pi_1)/m + (k_0^2\pi_0^2 + k_1^2\pi_1^2)}{(\pi_0 - \pi_1)^2}$$

where k_0 and k_1 are the between-cluster coefficients of variation of the true proportions in the control and intervention arms, respectively. If the intervention has a similar proportional effect across clusters,

then we can assume (as for event rates) that k_0 and k_1 have a common value k, and equation (8.6) simplifies to:

$$c = 1 + \left(z_{\alpha/2} + z_\beta\right)^2 \frac{\pi_0(1-\pi_0)/m + \pi_1(1-\pi_1)/m + k^2(\pi_0^2 + \pi_1^2)}{(\pi_0 - \pi_1)^2}$$

Note that the two previous formulas include a penalty of one additional cluster per arm to account for the fact that we are not in the "large sample" setting. To further protect against this, we take the more conservative penalty of adding an additional 2 clusters per arm, as is recommended by Hayes and Moulton for pair-matched designs. As such, Equation (8.6) becomes:

$$c = 2 + \left(z_{\alpha/2} + z_\beta\right)^2 \frac{\pi_0(1-\pi_0)/m + \pi_1(1-\pi_1)/m + k^2(\pi_0^2 + \pi_1^2)}{(\pi_0 - \pi_1)^2}$$

Table S1 Estimation of the intraclass correlation coefficients (ICCs) for the primary outcome

**Estimation of ICC based on original assumptions**					
**Country**	**Expected proportion of ACT users with positive test in control arm**	**95% confidence interval range of outcome across outlets**	**SD (i.e. approx quarter of 95% confidence interval range)**	**CV = SD/expected proportion in control**	**Estimated ICC**
Kenya	0.12	0.12	0.030	0.25	0.009
Nigeria	0.15	0.11	0.028	0.18	0.006
**Estimation of ICC based on pilot data**					
**Country**	**Expected proportion of ACT users with positive test in control arm**	**95% confidence interval range of outcome across outlets**	**SD (i.e. approx quarter of 95% confidence interval range)**	**CV = SD/expected proportion in control**	**Estimated ICC**
Kenya	0.07	0.06	0.015	0.22	0.003
Nigeria	0.21	0.18	0.045	0.21	0.01

Note: Intraclass correlation coefficients (ICCs) calculated using the formula ICC = k^2 * pi/(1-pi) where k=coefficient of variation (CV) and pi is the expected proportion of the outcome in the control arm [1]. To estimate the CVs, we assumed the outlet-specific proportions of the primary outcome to be normally distributed, centered on the control arm proportion with standard deviation (SD) derived from an assumed range for 95% of the outlet-specific proportions (e.g., for a width of ~4SD). To be conservative in power calculation, we chose the higher ICC estimated from either the original assumptions or the pilot data.

Table S2 Original assumptions about testing/ACT uptake and primary outcome in Kenya

**ASSUMPTIONS ABOUT TESTING/ACT UPTAKE**								
	**Control**		**PD (provider directed intervention)**		**CD (client directed intervention)**		**PD+CD (combined interventions)**	
	**%**	**N/cluster**	**%**	**N/cluster**	**%**	**N/cluster**	**%**	**N/cluster**
Fevers tested	30%	51	35%	59.5	35%	59.5	60%	102
Tested fevers that are positive	30%	15.3	30%	17.85	30%	17.85	30%	30.6
Positive fevers that take ACT	70%	10.71	70%	12.495	95%	16.9575	95%	29.07
Negative fevers that take ACT	30%	10.71	30%	12.495	20%	8.33	20%	14.28
Untested fevers that take ACT	60%	71.4	60%	66.3	60%	66.3	60%	40.8
Positive fevers that take non-ACT Am	30%	4.59	30%	5.355	5%	0.8925	5%	1.53
Negative fevers that take non-ACT Am	60%	21.42	60%	24.99	60%	24.99	60%	42.84
Untested fevers that take non-ACT Am	20%	23.8	20%	22.1	20%	22.1	20%	13.6

PRIMARY OUTCOME								
	Control		PD (provider directed intervention)		CD (client directed intervention)		PD+CD (combined interventions)	
	%	N (denom)	%	N (denom)	%	N (denom)	%	N (denom)
**% of ACTs used by positives**	12%	93	14%	91	19%	92	35%	84.15

Table S3 Original assumptions about testing/ACT uptake and primary outcome in Nigeria

ASSUMPTIONS ABOUT TESTING/ACT UPTAKE								
	Control		PD (provider directed intervention)		CD (client directed intervention)		PD+CD (combined interventions)	
	%	N/cluster	%	N/cluster	%	N/cluster	%	N/cluster
Fevers tested	55%	93.5	60%	102	60%	102	85%	144.5
Tested fevers that are positive	17%	15.895	17%	17.34	17%	17.34	17%	24.565
Positive fevers that take ACT	65%	10.33175	65%	11.271	95%	16.473	95%	23.33675
Negative fevers that take ACT	30%	23.2815	30%	25.398	20%	16.932	20%	23.987
Untested fevers that take ACT	45%	34.425	45%	30.6	45%	30.6	45%	11.475
Positive fevers that take non-ACT Am	27%	4.29165	27%	4.6818	5%	0.867	5%	1.22825
Negative fevers that take non-ACT Am	30%	23.2815	30%	25.398	30%	25.398	30%	35.9805
Untested fevers that take non-ACT Am	48%	36.72	48%	32.64	48%	32.64	48%	12.24
**PRIMARY OUTCOME**								
	**Control**		**PD (provider directed intervention)**		**CD (client directed intervention)**		**PD+CD (combined interventions)**	
	**%**	**N (denom)**	**%**	**N (denom)**	**%**	**N (denom)**	**%**	**N (denom)**
**% of ACTs used by positives**	15%	68	17%	67	26%	64	40%	58.79875

Table S4 Expected effect sizes for the main comparisons of interest in the primary outcome and sample size required for 90% power to detect those effect sizes based on original assumptions

**Primary Outcome Comparison**	**Kenya**		**Nigeria**	
	Expected Effect Size (change in percentage of ACTs taken by clients with a positive test)	Client Interviews required per cluster, assuming 40 clusters, for 90% power (alpha=0.05/3 = 0.0167)	Expected Effect Size (change in percentage of ACTs taken by those with a positive test)	Client Interviews required per cluster, assuming 48 clusters, for 90% power (alpha=0.05/3=0.0167)
Combined Interventions (PD+CD) vs. Control Arm	35% (PD+CD) – 12% (Control) = **23 percentage points**	30	40% (PD+CD) – 15% (Control) = **25 percentage points**	28
Combined Interventions (PD+CD) vs. Provider Directed Intervention (PD)	35% (PD+CD) – 14% (PD) = **21 percentage points**	43	40% (PD+CD) – 17% (PD) = **23 percentage points**	34
Combined Interventions (PD+CD) vs. Client Directed Intervention (CD)	35% (PD+CD) – 19% (CD) = **16 percentage points**	160	40% (PD+CD) – 26% (CD) = **14 percentage points**	176

Note: Two-tailed Type 1 error set at 0.0167 (0.05/3), and using standard methods for two-sample comparison of proportions, with adjustments for correlation of outcomes within clusters (see Table S1).

Table S5 Assumptions about testing/ACT uptake and primary outcome in Kenya based on pilot data

**ASSUMPTIONS ABOUT TESTING/ACT UPTAKE**								
	**Control**		**PD (provider directed intervention)**		**CD (client directed intervention)**		**PD+CD (combined interventions)**	
	**%**	**N/cluster**	**%**	**N/cluster**	**%**	**N/cluster**	**%**	**N/cluster**
Fevers tested	30%	51	35%	59.5	35%	59.5	60%	102
Tested fevers that are positive	20%	10.2	20%	11.9	20%	11.9	20%	20.4
Positive fevers that take ACT	70%	7.14	70%	8.33	95%	11.305	95%	19.38
Negative fevers that take ACT	30%	12.24	30%	14.28	20%	9.52	20%	16.32
Untested fevers that take ACT	70%	83.3	70%	77.35	70%	77.35	70%	47.6

Positive fevers that take non-ACT Am	30%	3.06	30%	3.57	5%	0.595	5%	1.02
Negative fevers that take non-ACT Am	20%	8.16	20%	9.52	20%	9.52	20%	16.32
Untested fevers that take non-ACT Am	20%	23.8	20%	22.1	20%	22.1	20%	13.6
**PRIMARY OUTCOME**								
	**Control**		**PD (provider directed intervention)**		**CD (client directed intervention)**		**PD+CD (combined interventions)**	
	**%**	**N (denom)**	**%**	**N (denom)**	**%**	**N (denom)**	**%**	**N (denom)**
**% of ACTs used by positives**	7%	103	8%	100	12%	98	23%	83.3

Table S6 Expected effect sizes for the main comparisons of interest in the primary outcome and power to detect those effect sizes in Kenya based on pilot data

	**Power based on original assumption**		**Power based on pilot data**			
**Primary Outcome Comparison**	**4-arm design** 10 clusters per arm (alpha=0.05/3 = 0.0167)		**4-arm design** 10 clusters per arm (alpha=0.05/3 = 0.0167)		**3-arm design** 13 clusters per arm (alpha=0.05/2=0.025)	
	Expected Effect Size	Power	Expected Effect Size	Power	Expected Effect Size	Power
Combined Interventions (PD+CD) vs. Control Arm	35% (PD+CD) – 12% (Control) = **23 percentage points**	100%	23% (PD+CD) – 7% (Control) = **16 percentage points**	93.6%	23% (PD+CD) – 7% (Control) = **16 percentage points**	99.1%
Combined Interventions (PD+CD) vs. Provider Directed Intervention (PD)	35% (PD+CD) – 14% (PD) = **21 percentage points**	99.7%	23% (PD+CD) – 8% (PD) = **15 percentage points**	86.9%	NA	NA
Combined Interventions (PD+CD) vs. Client Directed Intervention (CD)	35% (PD+CD) – 19% (CD) = **16 percentage points**	90.7%	23% (PD+CD) – 12% (CD) = **11 percentage points**	59.5%	23% (PD+CD) – 12% (CD) = **11 percentage points**	80.2%

Note: Expected effect size, change in percentage of ACTs taken by clients with a positive test. Since our outcome is a composite measure of testing rates and adherence to the test result, our sample size calculations accounted for the fact that not everyone who we interview will have taken an ACT.

Table S7 Assumptions about testing/ACT uptake and primary outcome in Nigeria based on pilot data

**ASSUMPTIONS ABOUT TESTING/ACT UPTAKE**								
	**Control**		**PD (provider directed intervention)**		**CD (client directed intervention)**		**PD+CD (combined interventions)**	
	**%**	**N/cluster**	**%**	**N/cluster**	**%**	**N/cluster**	**%**	**N/cluster**
Fevers tested	55%	93.5	60%	102	60%	102	85%	144.5
Tested fevers that are positive	17%	15.895	17%	17.34	17%	17.34	17%	24.565
Positive fevers that take ACT	58%	9.2191	58%	10.0572	88%	15.2592	88%	21.6172
Negative fevers that take ACT	9%	6.98445	9%	7.6194	1%	0.8466	1%	1.19935
Untested fevers that take ACT	35%	26.775	35%	23.8	35%	23.8	35%	8.925
Positive fevers that take non-ACT Am	32%	5.0864	32%	5.5488	10%	1.734	10%	2.4565
Negative fevers that take non-ACT Am	8%	6.2084	8%	6.7728	8%	6.7728	8%	9.5948
Untested fevers that take non-ACT Am	55%	42.075	55%	37.4	55%	37.4	55%	14.025
**PRIMARY OUTCOME**								
	**Control**		**PD (provider directed intervention)**		**CD (client directed intervention)**		**PD+CD (combined interventions)**	
	**%**	**N (denom)**	**%**	**N (denom)**	**%**	**N (denom)**	**%**	**N (denom)**
**% of ACTs used by positives**	21%	43	24%	41	38%	40	68%	31.74155

Table S8 Expected effect sizes for the main comparisons of interest in the primary outcome and power to detect those effect sizes in Nigeria based on pilot data

	**Power based on original assumption**		**Power based on pilot data**	
**Primary Outcome Comparison**	**4-arm design**<br>12 clusters per arm (alpha=0.05/3 = 0.0167)		**4-arm design**<br>12 clusters per arm (alpha=0.05/3 = 0.0167)	
	Expected Effect Size	Power	Expected Effect Size	Power
Combined Interventions (PD+CD) vs. Control Arm	40% (PD+CD) – 15% (Control) =<br>**25 percentage points**	100%	68% (PD+CD) – 21% (Control) =<br>**47 percentage points**	100%
Combined Interventions (PD+CD) vs.	40% (PD+CD) – 17% (PD) =<br>**23 percentage points**	100%	68% (PD+CD) – 24% (PD) =<br>**44 percentage points**	100%

Provider Directed Intervention (PD)				
Combined Interventions (PD+CD) vs. Client Directed Intervention (CD)	40% (PD+CD) – 26% (CD) = **14 percentage points**	89.4%	68% (PD+CD) – 38% (CD) = **30 percentage points**	98.8%

Note: Expected effect size, change in percentage of ACTs taken by clients with a positive test. Since our outcome is a composite measure of testing rates and adherence to the test result, our sample size calculations accounted for the fact that not everyone who we interview will have taken an ACT.

References

1. Hayes RJ, Moulton LH. Cluster randomised trials, second edition. Clust. Randomised Trials, Second Ed. 2017.
2. Aickin M, Gensler H. Adjusting for multiple testing when reporting research results: The Bonferroni vs Holm methods. Am J Public Health. 1996.
